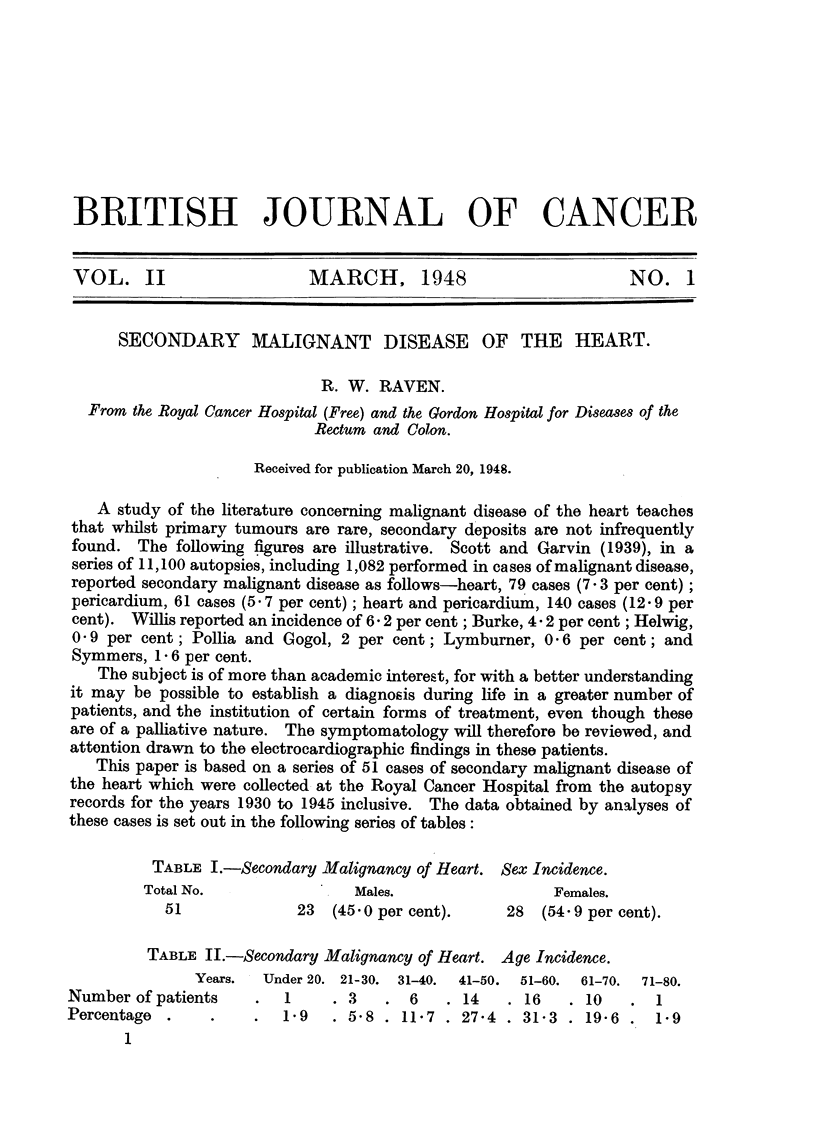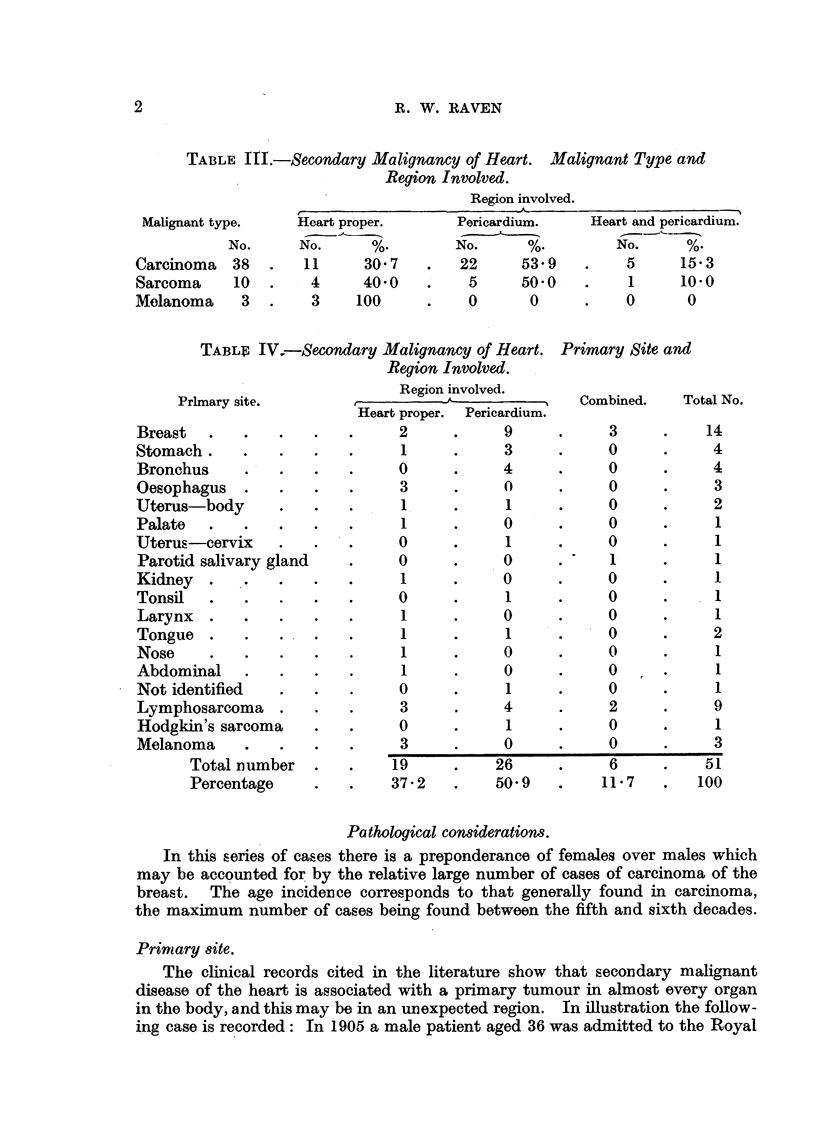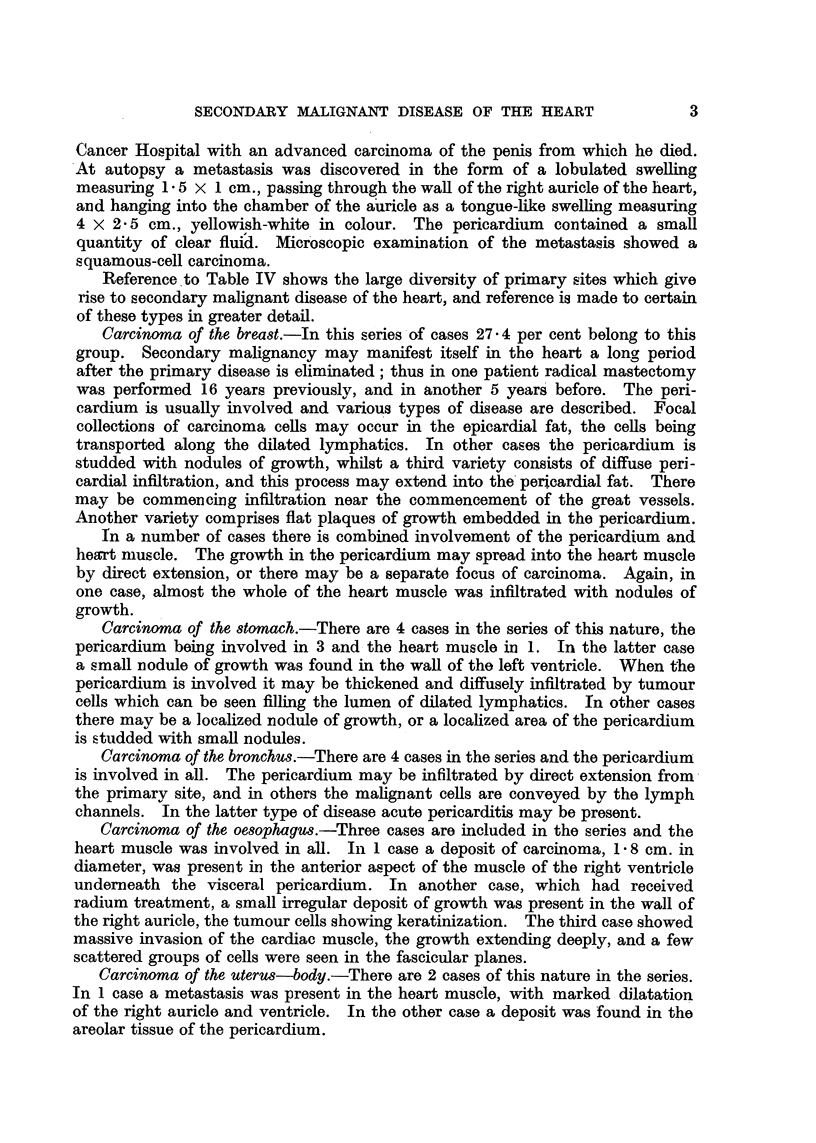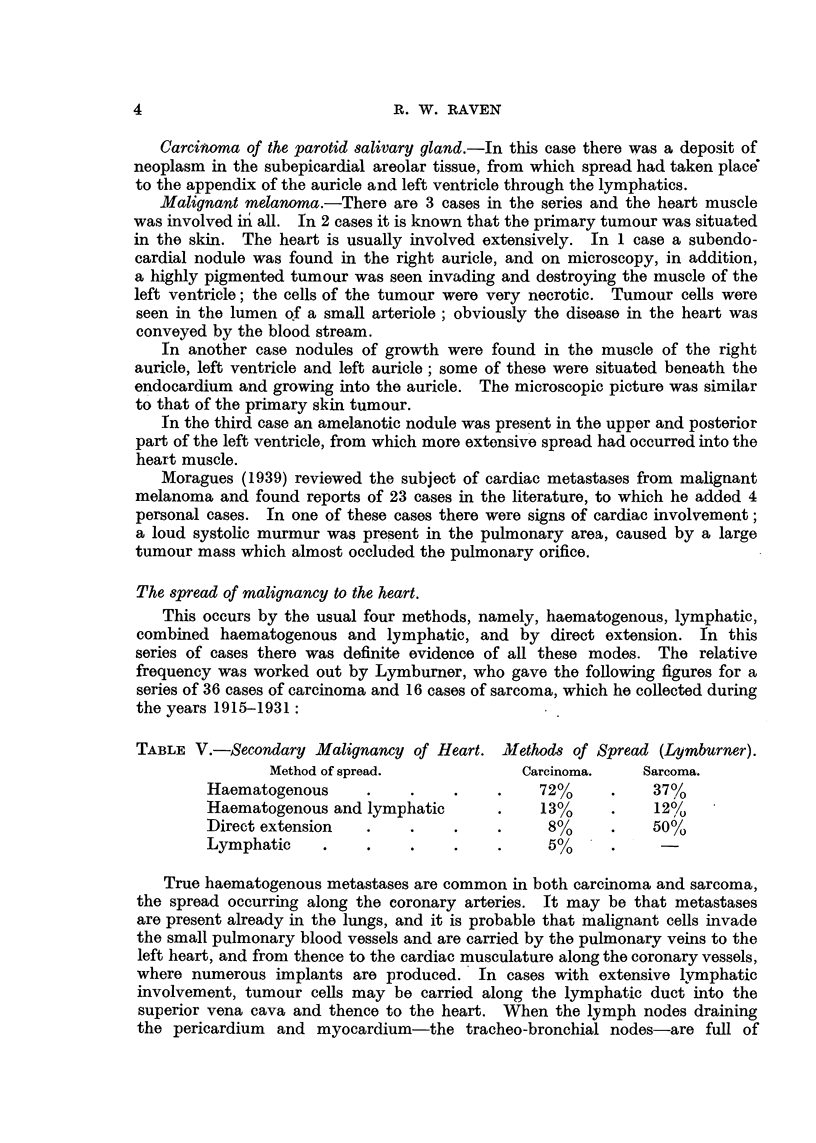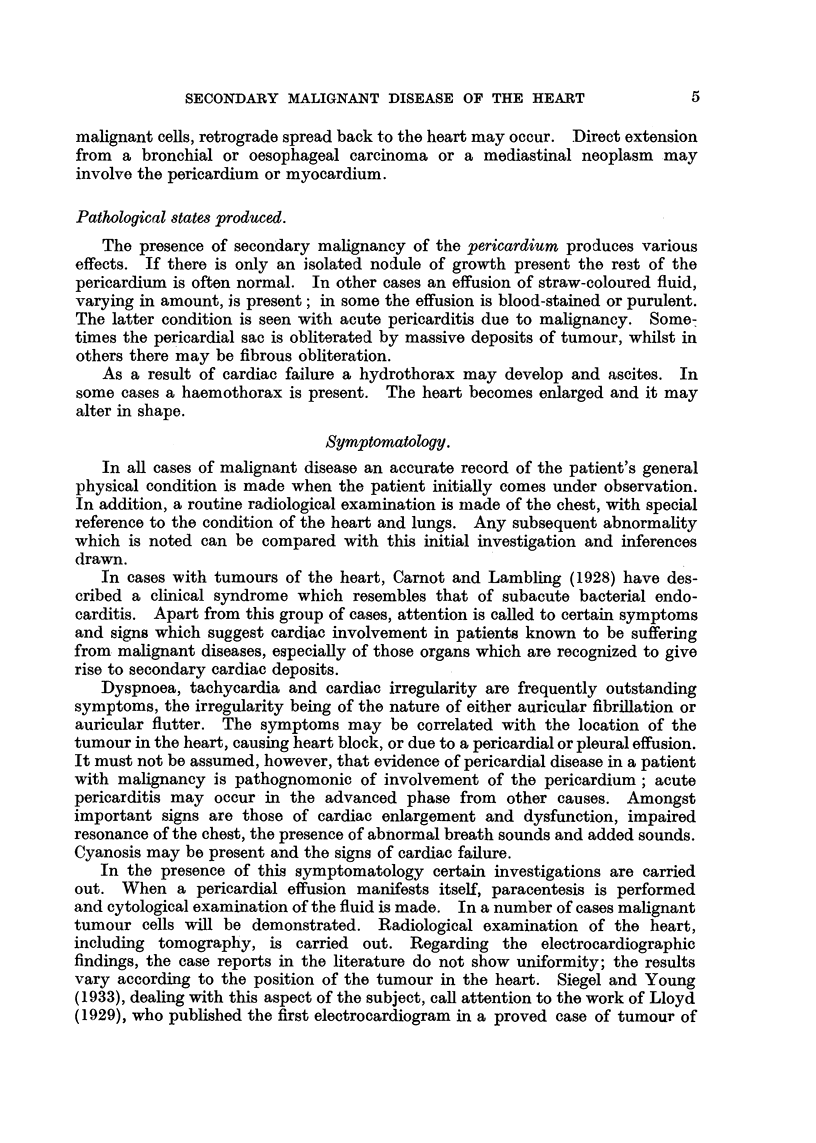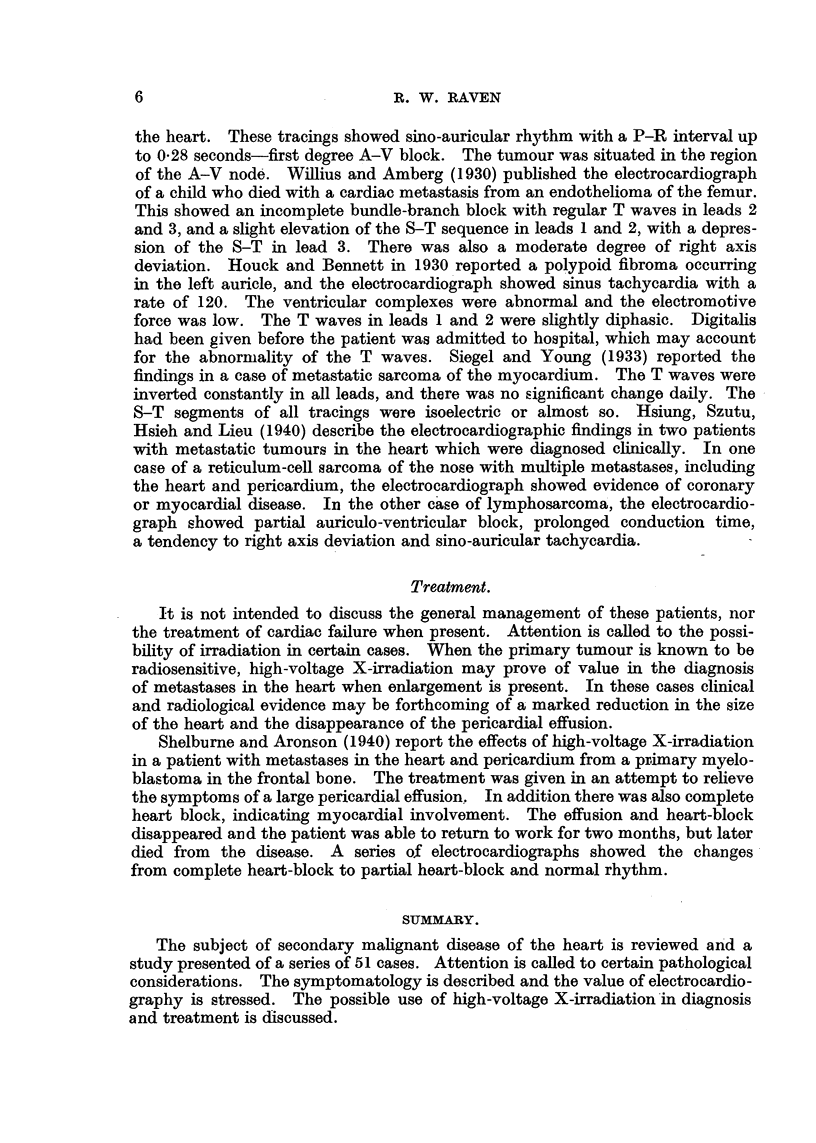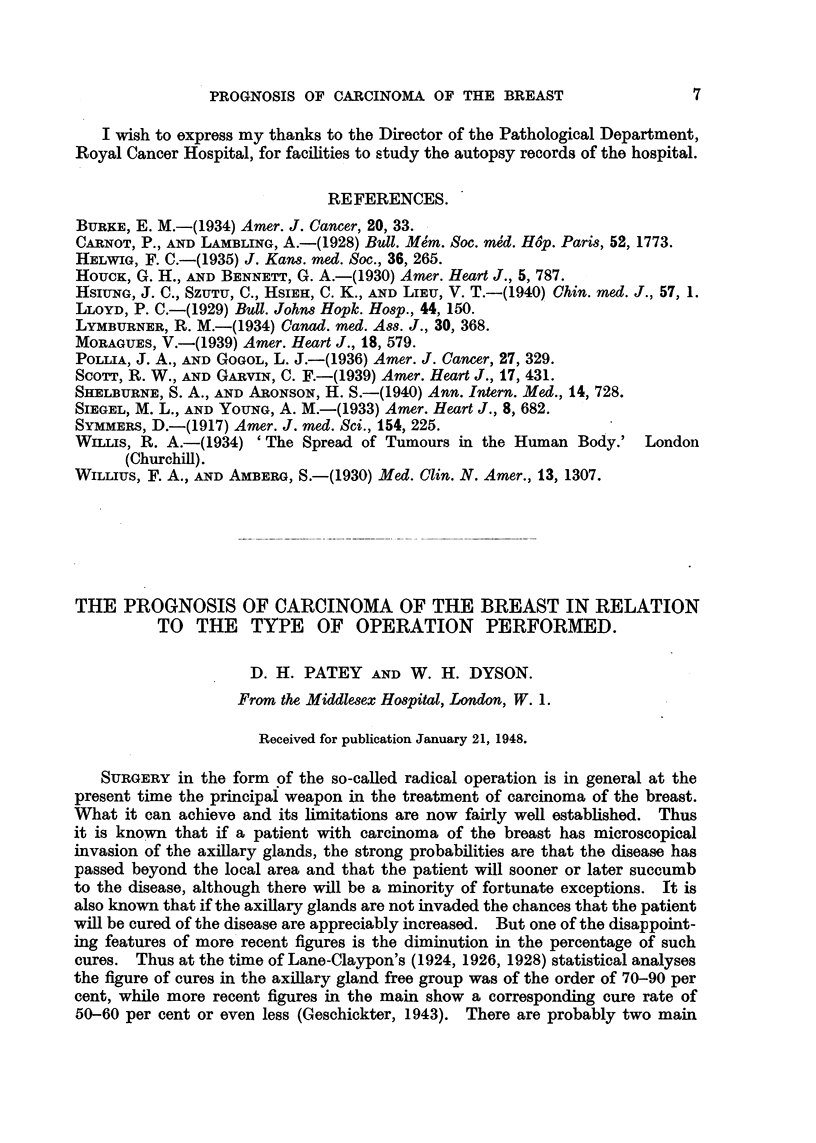# Secondary Malignant Disease of the Heart

**DOI:** 10.1038/bjc.1948.1

**Published:** 1948-03

**Authors:** R. W. Raven


### BRITISH JOURNAL OF CANCER

VOL. II MARCH, 1948 NO. 1

### SECONDARY MALIGNANT DISEASE OF THE HEART.

R. W. RAVEN.

*From the Royal Cancer Hospital (Free) and the Gordon Hospital for Diseases of the Rectum and Colon.*

Received for publication March 20, 1948.

A study of the literature concerning malignant disease of the heart teaches that whilst primary tumours are rare, secondary deposits are not infrequently found. The following figures are illustrative. Scott and Garvin (1939), in a series of 11,100 autopsies, including 1,082 performed in cases of malignant disease, reported secondary malignant disease as follows—heart, 79 cases (7·3 per cent); pericardium, 61 cases (5·7 per cent); heart and pericardium, 140 cases (12·9 per cent). Willis reported an incidence of 6·2 per cent; Burke, 4·2 per cent; Helwig, 0·9 per cent; Pollia and Gogol, 2 per cent; Lymburner, 0·6 per cent; and Symmers, 1·6 per cent.

The subject is of more than academic interest, for with a better understanding it may be possible to establish a diagnosis during life in a greater number of patients, and the institution of certain forms of treatment, even though these are of a palliative nature. The symptomatology will therefore be reviewed, and attention drawn to the electrocardiographic findings in these patients.

This paper is based on a series of 51 cases of secondary malignant disease of the heart which were collected at the Royal Cancer Hospital from the autopsy records for the years 1930 to 1945 inclusive. The data obtained by analyses of these cases is set out in the following series of tables:

TABLE I.—*Secondary Malignancy of Heart. Sex Incidence.*

| Total No. | Males. | Females. |
|---|---|---|
| 51 | 23 (45·0 per cent). | 28 (54·9 per cent). |

TABLE II.—*Secondary Malignancy of Heart. Age Incidence.*

| Years. | Under 20. | 21-30. | 31-40. | 41-50. | 51-60. | 61-70. | 71-80. |
|---|---|---|---|---|---|---|---|
| Number of patients | 1 | 3 | 6 | 14 | 16 | 10 | 1 |
| Percentage | 1·9 | 5·8 | 11·7 | 27·4 | 31·3 | 19·6 | 1·9 |

TABLE III.—*Secondary Malignancy of Heart. Malignant Type and Region Involved.*

| Malignant type. | | Region involved. Heart proper. | | Pericardium. | | Heart and pericardium. | |
|---|---|---|---|---|---|---|---|
| | No. | No. | %. | No. | %. | No. | %. |
| Carcinoma | 38 | 11 | 30·7 | 22 | 53·9 | 5 | 15·3 |
| Sarcoma | 10 | 4 | 40·0 | 5 | 50·0 | 1 | 10·0 |
| Melanoma | 3 | 3 | 100 | 0 | 0 | 0 | 0 |

TABLE IV.—*Secondary Malignancy of Heart. Primary Site and Region Involved.*

| Primary site. | Region involved. Heart proper. | Pericardium. | Combined. | Total No. |
|---|---|---|---|---|
| Breast | 2 | 9 | 3 | 14 |
| Stomach | 1 | 3 | 0 | 4 |
| Bronchus | 0 | 4 | 0 | 4 |
| Oesophagus | 3 | 0 | 0 | 3 |
| Uterus—body | 1 | 1 | 0 | 2 |
| Palate | 1 | 0 | 0 | 1 |
| Uterus—cervix | 0 | 1 | 0 | 1 |
| Parotid salivary gland | 0 | 0 | 1 | 1 |
| Kidney | 1 | 0 | 0 | 1 |
| Tonsil | 0 | 1 | 0 | 1 |
| Larynx | 1 | 0 | 0 | 1 |
| Tongue | 1 | 1 | 0 | 2 |
| Nose | 1 | 0 | 0 | 1 |
| Abdominal | 1 | 0 | 0 | 1 |
| Not identified | 0 | 1 | 0 | 1 |
| Lymphosarcoma | 3 | 4 | 2 | 9 |
| Hodgkin's sarcoma | 0 | 1 | 0 | 1 |
| Melanoma | 3 | 0 | 0 | 3 |
| Total number | 19 | 26 | 6 | 51 |
| Percentage | 37·2 | 50·9 | 11·7 | 100 |

*Pathological considerations.*

In this series of cases there is a preponderance of females over males which may be accounted for by the relative large number of cases of carcinoma of the breast. The age incidence corresponds to that generally found in carcinoma, the maximum number of cases being found between the fifth and sixth decades.

*Primary site.*

The clinical records cited in the literature show that secondary malignant disease of the heart is associated with a primary tumour in almost every organ in the body, and this may be in an unexpected region. In illustration the following case is recorded: In 1905 a male patient aged 36 was admitted to the Royal

Cancer Hospital with an advanced carcinoma of the penis from which he died. At autopsy a metastasis was discovered in the form of a lobulated swelling measuring $1 \cdot 5 \times 1$ cm., passing through the wall of the right auricle of the heart, and hanging into the chamber of the auricle as a tongue-like swelling measuring $4 \times 2 \cdot 5$ cm., yellowish-white in colour. The pericardium contained a small quantity of clear fluid. Microscopic examination of the metastasis showed a squamous-cell carcinoma.

Reference to Table IV shows the large diversity of primary sites which give rise to secondary malignant disease of the heart, and reference is made to certain of these types in greater detail.

*Carcinoma of the breast.*—In this series of cases 27·4 per cent belong to this group. Secondary malignancy may manifest itself in the heart a long period after the primary disease is eliminated; thus in one patient radical mastectomy was performed 16 years previously, and in another 5 years before. The pericardium is usually involved and various types of disease are described. Focal collections of carcinoma cells may occur in the epicardial fat, the cells being transported along the dilated lymphatics. In other cases the pericardium is studded with nodules of growth, whilst a third variety consists of diffuse pericardial infiltration, and this process may extend into the pericardial fat. There may be commencing infiltration near the commencement of the great vessels. Another variety comprises flat plaques of growth embedded in the pericardium.

In a number of cases there is combined involvement of the pericardium and heart muscle. The growth in the pericardium may spread into the heart muscle by direct extension, or there may be a separate focus of carcinoma. Again, in one case, almost the whole of the heart muscle was infiltrated with nodules of growth.

*Carcinoma of the stomach.*—There are 4 cases in the series of this nature, the pericardium being involved in 3 and the heart muscle in 1. In the latter case a small nodule of growth was found in the wall of the left ventricle. When the pericardium is involved it may be thickened and diffusely infiltrated by tumour cells which can be seen filling the lumen of dilated lymphatics. In other cases there may be a localized nodule of growth, or a localized area of the pericardium is studded with small nodules.

*Carcinoma of the bronchus.*—There are 4 cases in the series and the pericardium is involved in all. The pericardium may be infiltrated by direct extension from the primary site, and in others the malignant cells are conveyed by the lymph channels. In the latter type of disease acute pericarditis may be present.

*Carcinoma of the oesophagus.*—Three cases are included in the series and the heart muscle was involved in all. In 1 case a deposit of carcinoma, 1·8 cm. in diameter, was present in the anterior aspect of the muscle of the right ventricle underneath the visceral pericardium. In another case, which had received radium treatment, a small irregular deposit of growth was present in the wall of the right auricle, the tumour cells showing keratinization. The third case showed massive invasion of the cardiac muscle, the growth extending deeply, and a few scattered groups of cells were seen in the fascicular planes.

*Carcinoma of the uterus—body.*—There are 2 cases of this nature in the series. In 1 case a metastasis was present in the heart muscle, with marked dilatation of the right auricle and ventricle. In the other case a deposit was found in the areolar tissue of the pericardium.

*Carcinoma of the parotid salivary gland.*—In this case there was a deposit of neoplasm in the subepicardial areolar tissue, from which spread had taken place to the appendix of the auricle and left ventricle through the lymphatics.

*Malignant melanoma.*—There are 3 cases in the series and the heart muscle was involved in all. In 2 cases it is known that the primary tumour was situated in the skin. The heart is usually involved extensively. In 1 case a subendocardial nodule was found in the right auricle, and on microscopy, in addition, a highly pigmented tumour was seen invading and destroying the muscle of the left ventricle; the cells of the tumour were very necrotic. Tumour cells were seen in the lumen of a small arteriole ; obviously the disease in the heart was conveyed by the blood stream.

In another case nodules of growth were found in the muscle of the right auricle, left ventricle and left auricle ; some of these were situated beneath the endocardium and growing into the auricle. The microscopic picture was similar to that of the primary skin tumour.

In the third case an amelanotic nodule was present in the upper and posterior part of the left ventricle, from which more extensive spread had occurred into the heart muscle.

Moragues (1939) reviewed the subject of cardiac metastases from malignant melanoma and found reports of 23 cases in the literature, to which he added 4 personal cases. In one of these cases there were signs of cardiac involvement; a loud systolic murmur was present in the pulmonary area, caused by a large tumour mass which almost occluded the pulmonary orifice.

*The spread of malignancy to the heart.*

This occurs by the usual four methods, namely, haematogenous, lymphatic, combined haematogenous and lymphatic, and by direct extension. In this series of cases there was definite evidence of all these modes. The relative frequency was worked out by Lymburner, who gave the following figures for a series of 36 cases of carcinoma and 16 cases of sarcoma, which he collected during the years 1915–1931 :

TABLE V.—*Secondary Malignancy of Heart. Methods of Spread (Lymburner).*

| Method of spread. | Carcinoma. | Sarcoma. |
|---|---|---|
| Haematogenous | 72% | 37% |
| Haematogenous and lymphatic | 13% | 12% |
| Direct extension | 8% | 50% |
| Lymphatic | 5% | — |

True haematogenous metastases are common in both carcinoma and sarcoma, the spread occurring along the coronary arteries. It may be that metastases are present already in the lungs, and it is probable that malignant cells invade the small pulmonary blood vessels and are carried by the pulmonary veins to the left heart, and from thence to the cardiac musculature along the coronary vessels, where numerous implants are produced. In cases with extensive lymphatic involvement, tumour cells may be carried along the lymphatic duct into the superior vena cava and thence to the heart. When the lymph nodes draining the pericardium and myocardium—the tracheo-bronchial nodes—are full of malignant cells, retrograde spread back to the heart may occur. Direct extension from a bronchial or oesophageal carcinoma or a mediastinal neoplasm may involve the pericardium or myocardium.

*Pathological states produced.*

The presence of secondary malignancy of the *pericardium* produces various effects. If there is only an isolated nodule of growth present the rest of the pericardium is often normal. In other cases an effusion of straw-coloured fluid, varying in amount, is present; in some the effusion is blood-stained or purulent. The latter condition is seen with acute pericarditis due to malignancy. Sometimes the pericardial sac is obliterated by massive deposits of tumour, whilst in others there may be fibrous obliteration.

As a result of cardiac failure a hydrothorax may develop and ascites. In some cases a haemothorax is present. The heart becomes enlarged and it may alter in shape.

*Symptomatology.*

In all cases of malignant disease an accurate record of the patient's general physical condition is made when the patient initially comes under observation. In addition, a routine radiological examination is made of the chest, with special reference to the condition of the heart and lungs. Any subsequent abnormality which is noted can be compared with this initial investigation and inferences drawn.

In cases with tumours of the heart, Carnot and Lambling (1928) have described a clinical syndrome which resembles that of subacute bacterial endocarditis. Apart from this group of cases, attention is called to certain symptoms and signs which suggest cardiac involvement in patients known to be suffering from malignant diseases, especially of those organs which are recognized to give rise to secondary cardiac deposits.

Dyspnoea, tachycardia and cardiac irregularity are frequently outstanding symptoms, the irregularity being of the nature of either auricular fibrillation or auricular flutter. The symptoms may be correlated with the location of the tumour in the heart, causing heart block, or due to a pericardial or pleural effusion. It must not be assumed, however, that evidence of pericardial disease in a patient with malignancy is pathognomonic of involvement of the pericardium; acute pericarditis may occur in the advanced phase from other causes. Amongst important signs are those of cardiac enlargement and dysfunction, impaired resonance of the chest, the presence of abnormal breath sounds and added sounds. Cyanosis may be present and the signs of cardiac failure.

In the presence of this symptomatology certain investigations are carried out. When a pericardial effusion manifests itself, paracentesis is performed and cytological examination of the fluid is made. In a number of cases malignant tumour cells will be demonstrated. Radiological examination of the heart, including tomography, is carried out. Regarding the electrocardiographic findings, the case reports in the literature do not show uniformity; the results vary according to the position of the tumour in the heart. Siegel and Young (1933), dealing with this aspect of the subject, call attention to the work of Lloyd (1929), who published the first electrocardiogram in a proved case of tumour of the heart. These tracings showed sino-auricular rhythm with a P–R interval up to 0·28 seconds—first degree A–V block. The tumour was situated in the region of the A–V node. Willius and Amberg (1930) published the electrocardiograph of a child who died with a cardiac metastasis from an endothelioma of the femur. This showed an incomplete bundle-branch block with regular T waves in leads 2 and 3, and a slight elevation of the S–T sequence in leads 1 and 2, with a depression of the S–T in lead 3. There was also a moderate degree of right axis deviation. Houck and Bennett in 1930 reported a polypoid fibroma occurring in the left auricle, and the electrocardiograph showed sinus tachycardia with a rate of 120. The ventricular complexes were abnormal and the electromotive force was low. The T waves in leads 1 and 2 were slightly diphasic. Digitalis had been given before the patient was admitted to hospital, which may account for the abnormality of the T waves. Siegel and Young (1933) reported the findings in a case of metastatic sarcoma of the myocardium. The T waves were inverted constantly in all leads, and there was no significant change daily. The S–T segments of all tracings were isoelectric or almost so. Hsiung, Szutu, Hsieh and Lieu (1940) describe the electrocardiographic findings in two patients with metastatic tumours in the heart which were diagnosed clinically. In one case of a reticulum-cell sarcoma of the nose with multiple metastases, including the heart and pericardium, the electrocardiograph showed evidence of coronary or myocardial disease. In the other case of lymphosarcoma, the electrocardiograph showed partial auriculo-ventricular block, prolonged conduction time, a tendency to right axis deviation and sino-auricular tachycardia.

*Treatment.*

It is not intended to discuss the general management of these patients, nor the treatment of cardiac failure when present. Attention is called to the possibility of irradiation in certain cases. When the primary tumour is known to be radiosensitive, high-voltage X-irradiation may prove of value in the diagnosis of metastases in the heart when enlargement is present. In these cases clinical and radiological evidence may be forthcoming of a marked reduction in the size of the heart and the disappearance of the pericardial effusion.

Shelburne and Aronson (1940) report the effects of high-voltage X-irradiation in a patient with metastases in the heart and pericardium from a primary myeloblastoma in the frontal bone. The treatment was given in an attempt to relieve the symptoms of a large pericardial effusion. In addition there was also complete heart block, indicating myocardial involvement. The effusion and heart-block disappeared and the patient was able to return to work for two months, but later died from the disease. A series of electrocardiographs showed the changes from complete heart-block to partial heart-block and normal rhythm.

SUMMARY.

The subject of secondary malignant disease of the heart is reviewed and a study presented of a series of 51 cases. Attention is called to certain pathological considerations. The symptomatology is described and the value of electrocardiography is stressed. The possible use of high-voltage X-irradiation in diagnosis and treatment is discussed.

I wish to express my thanks to the Director of the Pathological Department, Royal Cancer Hospital, for facilities to study the autopsy records of the hospital.

### REFERENCES.

BURKE, E. M.—(1934) *Amer. J. Cancer*, **20**, 33.
CARNOT, P., AND LAMBLING, A.—(1928) *Bull. Mém. Soc. méd. Hôp. Paris*, **52**, 1773.
HELWIG, F. C.—(1935) *J. Kans. med. Soc.*, **36**, 265.
HOUCK, G. H., AND BENNETT, G. A.—(1930) *Amer. Heart J.*, **5**, 787.
HSIUNG, J. C., SZUTU, C., HSIEH, C. K., AND LIEU, V. T.—(1940) *Chin. med. J.*, **57**, 1.
LLOYD, P. C.—(1929) *Bull. Johns Hopk. Hosp.*, **44**, 150.
LYMBURNER, R. M.—(1934) *Canad. med. Ass. J.*, **30**, 368.
MORAGUES, V.—(1939) *Amer. Heart J.*, **18**, 579.
POLLIA, J. A., AND GOGOL, L. J.—(1936) *Amer. J. Cancer*, **27**, 329.
SCOTT, R. W., AND GARVIN, C. F.—(1939) *Amer. Heart J.*, **17**, 431.
SHELBURNE, S. A., AND ARONSON, H. S.—(1940) *Ann. Intern. Med.*, **14**, 728.
SIEGEL, M. L., AND YOUNG, A. M.—(1933) *Amer. Heart J.*, **8**, 682.
SYMMERS, D.—(1917) *Amer. J. med. Sci.*, **154**, 225.
WILLIS, R. A.—(1934) 'The Spread of Tumours in the Human Body.' London (Churchill).
WILLIUS, F. A., AND AMBERG, S.—(1930) *Med. Clin. N. Amer.*, **13**, 1307.

---